# Effects of Multisensory Environmental Cues on Food Craving, Healthy Food Preference, and Stress-Related Recovery

**DOI:** 10.3390/bs16091471

**Published:** 2026-08-24

**Authors:** Lu Zhang, Shulan Yu, Qi Li

**Affiliations:** 1 College of Furnishings and Industrial Design, Nanjing Forestry University, Nanjing 210037, China; zhanglu0214@njfu.edu.cn; 2 Beijing Key Laboratory of Learning and Cognition, Department of Psychology, Capital Normal University, Beijing 100048, China

**Keywords:** emotional eating, food preference, food craving, virtual reality (VR), multisensory environment

## Abstract

Emotional eating (EE) is associated with poor dietary quality and weight gain among university students. Therefore, exploring innovative, non-invasive interventions to support healthier food-related responses is essential. While multisensory environments show promise in influencing food-related responses, their effectiveness in managing stress-induced EE remains understudied. Grounded in the Environment–Organism–Health (EOH) model, this study used an immersive virtual reality (VR) platform and recruited 49 Chinese university students. After emotional induction tasks, participants were randomly assigned to VR campus environments with varying wall colors and environmental sounds. The study combined physiological indicators and subjective questionnaires to evaluate the effects of color–sound combinations on emotional responses, physiological recovery, food preferences, and food cravings. Food preference was assessed via an image-selection task, and food healthiness was rated by an expert panel (*n* = 7). A mixed-design analysis of variance (ANOVA) revealed a significant interaction between wall color and sound. Natural sounds were associated with reduced negative emotions and higher healthy food preference scores under several color conditions, while both color and sound influenced food craving. Independent *t*-tests revealed significant differences according to gender and emotional eating tendency on multiple outcomes. This study suggests that multisensory environments may offer a novel approach to influencing stress-related emotional responses, food preferences, and food cravings.

## 1. Introduction

In recent years, the mental health issues faced by college students have become increasingly severe. Negative emotions such as anxiety, depression, and chronic stress have become key factors affecting their academic performance and quality of life (Shah et al., 2025). Under the combined impact of academic pressure, interpersonal conflicts, and uncertain career prospects, college students who lack effective stress management strategies often exhibit a series of maladaptive coping behaviors (Abdella et al., 2019; Marchena-Giraldez et al., 2024). Among these, Emotional Eating (EE), a typical form of maladaptive emotional regulation, is gaining increasing attention in mental health research. Furthermore, studies show that negative emotion-related eating behaviors are prevalent among urban college students in China; however, there is still a lack of systematic identification and intervention mechanisms (Sze et al., 2021).

Emotional Eating refers to a non-homeostatic eating behavior that occurs in a negative emotional state of stress or anxiety and is not based on physiological hunger (Houminer Klepar et al., 2024). Studies have shown that negative emotions are one of the important inducements of emotional eating behavior (Barnhart et al., 2023; Rodriguez & Kross, 2023). When individuals are anxious, stressed, or feeling down, they are more likely to seek temporary emotional relief by consuming high-sugar and high-fat foods (Ha & Lim, 2023; Stammers et al., 2020). At the physiological level, when individuals experience stress, the hypothalamic–pituitary–adrenal (HPA) axis is activated, leading to elevated cortisol levels that trigger cravings for energy-dense, high-calorie foods (Warren & Frame, 2025). Specifically, stressed individuals tend to prefer “comfort foods”—typically high in sugar, fat, and salt—as a form of emotional self-medication, as these foods can temporarily activate reward pathways in the brain and alleviate negative affect (Klatzkin et al., 2024). Several empirical studies have further confirmed that EE is significantly correlated with eating disorders, and prolonged engagement in this behavior pattern can lead to excessive energy intake, weight gain, and the risks of obesity and metabolic health issues (Barnhart et al., 2024; Braden et al., 2025). Research indicates that emotional eating serves as a mediating factor between perceived stress and unhealthy food intake among university students, with downstream effects on dietary quality and long-term health (Ling & Zahry, 2021). 

While clinical interventions such as cognitive behavioral therapy have shown effectiveness for individuals with diagnosed eating disorders, less attention has been given to non-clinical, stress-induced emotional eating in general populations (Nicolau et al., 2022; van Beers et al., 2024). Recent research has increasingly focused on non-pharmacological, environmental, and behavioral interventions—such as mindfulness-based approaches and sensory environment modifications—as accessible strategies for managing everyday emotional eating behaviors (Daniela et al., 2021; Grider et al., 2021). These perspectives align with a behavioral change approach that emphasizes modifiable environmental factors rather than medical interventions. Therefore, this study explores whether multisensory environmental cues can serve as an accessible, non-invasive strategy to modulate stress-induced eating behaviors among college students.

Environmental interventions at the perceptual level, as a non-invasive and context-friendly approach, are gaining attention. Research indicates that multi-channel sensory stimuli from the external environment, such as colors, sounds, and scents, can influence an individual’s impulse and control over food by activating the brain’s reward system (Palazzo et al., 2022). Colors, as primary visual information, not only evoke various emotional experiences but also influence attention and motivation, thereby modulating individuals’ food response tendencies (Xia et al., 2023). Furthermore, indoor colors not only evoke specific emotional responses but also influence arousal levels, attention allocation, and cognitive assessments, which in turn affect motivation and behavioral tendencies (X. Gao et al., 2025; Yu et al., 2021). For example, warm-toned colors like red and orange typically evoke feelings of excitement, alertness, or tension, whereas cool-toned colors such as blue and green are associated with calmness and relaxation (Alvarado, 2025). Neuroimaging studies have further shown that colors can activate key areas of the brain’s reward system (such as the nucleus accumbens and prefrontal cortex), which are closely related to emotion regulation and eating motivation (Michels et al., 2021). Sound, as another significant environmental variable, has also been shown to effectively influence emotional states and eating behaviors (Qiu & Wan, 2024). Research indicates that natural sounds, such as the sound of a spring (Michels & Hamers, 2023; N. Zhang et al., 2025; R. Zhu et al., 2024), along with music environments of various rhythms and styles, can affect food intake, meal duration, and the selection of emotionally and healthily chosen foods (Mathiesen et al., 2022; Motoki et al., 2022). Additionally, growing research shows that environmental design, physical attributes, and individuals’ subjective perceptions of their surroundings significantly impact their immediate emotional state and behavioral responses (Li et al., 2025).

While both colors and sounds can independently influence emotions and behaviors, sensory stimuli in real-world environments are typically perceived and integrated in combination. In recent years, studies have begun to explore the application of multisensory environments in regulating eating behaviors, for example, by using color, music, and lighting to enhance dining enjoyment, increase food appeal, or guide healthier dietary choices in dining settings (Shu et al., 2025; Szocs et al., 2024). Research indicates that physical factors such as color, lighting, and sound in the campus environment not only influence an individual’s emotional recovery but may also unconsciously shape their behavioral patterns (Al Horr et al., 2016). Further evidence from real campus environments shows an interaction between visual and auditory stimuli, with spatial visual ambiance (such as warm or cool tones) and background music serving as critical factors influencing the emotional valence of university students (Jiang et al., 2021). Importantly, research utilizing VR multimodal platforms to recreate campus scenarios has validated the essential role of visual and auditory environments in the psychological stress recovery of university students (Xu et al., 2024).

Although studies have shown that external environmental cues, such as visual and auditory stimuli, can significantly influence an individual’s emotional state and eating motivation, most of these studies focus on eating behaviors in daily or positive emotional contexts, lacking systematic research on EE behaviors triggered by negative emotions (Lin et al., 2019). In terms of research methodology, current studies on EE primarily focus on internal mechanisms within individuals, often using psychological questionnaires or self-reports to assess EE tendencies. These studies mainly explore the psychological pathways between emotions, stress, and eating behavior, or examine the role of individual variables (such as body mass index and personality traits) in EE (Bozkurt et al., 2024; Marchena-Giraldez et al., 2024). Commonly used assessment tools in these studies include the Dutch Eating Behavior Questionnaire (DEBQ), the State–Trait Anxiety Inventory (STAI), and the Food Craving Questionnaire—State (FCQ-S). These tools are widely used to measure the extent of emotional eating, emotional fluctuations, and non-hunger eating motives, and they have good reliability and validity. Although the FCQ-S does not directly represent EE behavior, its elevated scores in negative emotional situations can be seen as a psychological signal indicating the activation of EE tendencies, particularly in individuals with high DEBQ-EES scores, indirectly reflecting the impact of environmental factors on the pathways related to EE (Ge et al., 2024; Nolan & Jenkins, 2019). Additionally, physiological indicators, including skin conductance level (SCL) and heart rate (HR), have been used to assess the stress recovery process in individuals, demonstrating their ability to reflect emotional arousal and reward mechanisms associated with EE. 

However, there is a lack of empirical frameworks that integrate external environmental factors, physiological indicators, and EE behaviors (Meng & Zhang, 2025). The various environmental–behavior models proposed by architectural psychology and environmental behavior science provide a theoretical foundation for this study. Zeisel et al. developed the Environmental–Behavior (E-B) model, which lays the groundwork for understanding how physical environments influence human behavior (Zeisel et al., 2003). On this basis, Zhang et al. proposed the Environment–Organism–Health (EOH) framework, which emphasized how indoor physical conditions affect the psychological and physiological state of residents and then health outcomes (Y. Zhang et al., 2018). Despite the mature application of these models in performance, attention, and health indicators, there is still insufficient focus on EE, a typical form of emotional regulation through dietary behavior (Sanes, 2019).

Traditional studies often face a trade-off between experimental control and real-world relevance: laboratory settings provide greater control over variables but lack ecological validity, while field studies offer ecological validity but limited experimental control. Virtual reality (VR) can help bridge this gap by providing immersive and controlled environments for investigating eating-related behaviors while maintaining a relatively naturalistic behavioral context (Rosa et al., 2026). 

Given the high levels of stress and emotional fluctuations among college students, who spend much of their daily time in indoor campus spaces, indoor environmental sensory attributes may influence their psychological and behavioral states (Fan et al., 2022; Wang et al., 2024). Therefore, this study, based on the EOH theoretical model, uses an immersive VR platform to create a multisensory campus virtual environment that integrates environmental colors and background sounds. It systematically explores how different combinations of color and sound elements affect college students’ emotional responses, physiological recovery efficiency, food craving levels, and preferences for healthy foods under stress (Harris et al., 2023). Based on the above theoretical framework, this study proposes the following research hypotheses:

**H1.**  *Visual (color) and auditory (sound) stimuli interact to influence stress recovery, with the effect of auditory stimuli expected to vary across visual color conditions.*

**H2.**  *Multisensory environmental conditions influence acute stress-induced food craving (measured by FCQ-S) and preferences for healthier food options.*

**H3.**  *Individual characteristics, including gender and emotional eating tendencies (DEBQ-EES), are associated with individual differences in emotional states and food craving following exposure to multisensory environmental conditions.*

## 2. Materials and Methods

### 2.1. Experimental Environment Setting

The VR experiment was conducted in a controlled university conference room. This space is commonly used for academic defenses and public presentations, which typically involve evaluative and social pressures, making it prone to eliciting psychological stress. This aligns with the concept of evaluative space in environmental psychology, known to reliably induce acute psychological stress through social evaluative threat and performance demands (Mehrabian & Russell, 1974). The Apple Vision Pro headset (Apple Inc., Cupertino, CA, USA) served as the virtual reality platform to present participants with virtual environments composed of various environmental elements (Wienrich & Obremski, 2025; X. Zhu et al., 2025).

**Figure 1:**
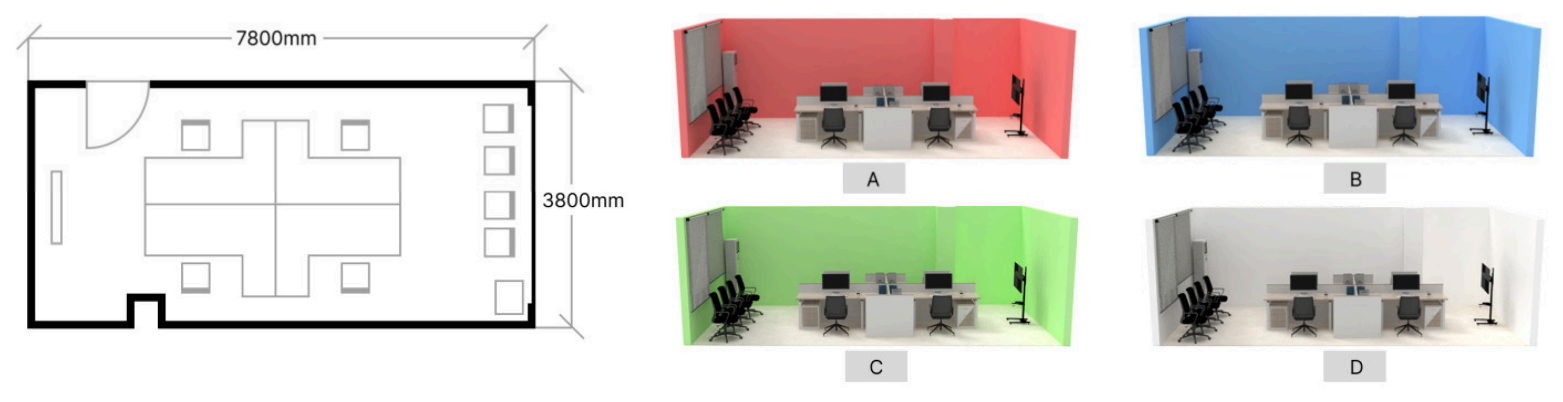
Schematic diagrams of the four experimental wall-color conditions. (**A**) Red. (**B**) Blue. (**C**) Green. (**D**) White.

The experiment utilized four representative wall colors, specifically red, green, blue, and white, to systematically induce varying degrees of emotional arousal (Figure 1). Prior studies have demonstrated that different hues can evoke distinct emotional responses even when brightness and saturation are held constant: red is associated with higher arousal and excitement, green with calm and relaxation, blue with tranquility and serenity, and white with neutrality and minimal emotional valence.

For the sound environment, considering that musical rhythm and style may introduce variability in emotional responses due to individual preferences and cultural backgrounds, the study selected spring water sounds and typical indoor background noises (such as soft conversation or keyboard typing sounds) as sound stimuli. All sound materials were sourced from a public emotional sound database and standardized to 60 dB. 

Before the experiment, the viewing perspective and color settings were calibrated. For the spatial environment construction, we first achieved 1:1 simulation modeling of spatial structure, materials, and furniture layout using Blender, and further optimized the scene for color, lighting, and audio integration in Xcode and Reality Composer Pro. 

During the experiment, participants interacted with the system using a standard wireless optical mouse, while the system recorded their response times and behavioral data. To monitor participants’ physiological states in real time, the study used the Ergosensing smart wearable wristband (Ergosensing Beijing, China), which continuously collected heart rate and skin conductance data during both resting and stress task phases. Additionally, the entire experimental procedure was video recorded for subsequent behavioral analysis and process review.

### 2.2. Study Design

This experiment was a mixed-design interventional study conducted at Nanjing Forestry University in Nanjing, China, from 10 May to 20 June 2025. A 4 (color) × 2 (sound) mixed design was employed, with sound (spring water sounds and indoor background sounds) as the within-subjects factor and color (red, green, blue, and white) as the between-subjects factor. Each participant was randomly assigned to one of the color conditions and experienced both sound conditions in a counterbalanced order (AB or BA; A: spring water sounds; B: indoor background sounds). Randomization resulted in approximately equal sample sizes across the color groups.

### 2.3. Participants

A total of 51 Chinese university students were recruited through advertisements and personal contacts. All participants were Han Chinese. Inclusion criteria: (1) age between 18 and 30 years; (2) currently enrolled as a student; (3) good general health, with no cold, fever, hearing impairment, color blindness, history of motion sickness, or other serious illnesses; (4) uncorrected or corrected visual acuity of ≥1.0; (5) no self-reported diagnosis of an eating disorder or psychiatric disorder; and (6) no use of medications affecting cardiovascular function. Exclusion criteria were failure to fast for at least 2 h before testing, VR-induced motion sickness, and incomplete physiological data. Using G*Power 3.1 with an effect size of 0.5, an *α* level of 0.05, and a power of 0.8, the required minimum sample size was calculated to be 48. Two participants were excluded due to incomplete physiological data, resulting in a final sample of 49 participants (age range = 22–28 years, *M* age = 22.43, *SD* = 3.19 years). Among the participants, 46.9% were female (*n* = 23) and 53.1% were male (*n* = 26), with most being undergraduate and graduate students.

The protocol was reviewed and approved by the Ethics Review Committee of Nanjing Forestry University (Approval No. 2025014). All participants provided informed consent before the experiment and received appropriate compensation.

### 2.4. Experimental Procedure

To control the effect of hunger on food craving ratings, all participants were required to fast for at least 2 h before the experiment. Research assistants confirmed the time of participants’ last meal before the experiment, and if it did not meet the required standard, the experiment was postponed, ensuring that food craving responses primarily reflected the induced effects under experimental conditions. In addition, to minimize the interference of biological rhythms on psychological and physiological states, the experiment was consistently scheduled between 14:00 and 17:00. 

Participants experienced two different environmental combination scenarios in a random order, and their emotional responses, physiological changes, and food preference data were systematically recorded under stress conditions. To enhance immersion and ensure experimental control, participants began the experiment seated and entered the virtual reality scene with the observation point fixed 1.5 m from the door of the virtual conference room. The spatial viewpoint was fixed to avoid attention shifts due to position differences. 

**Figure 2:**
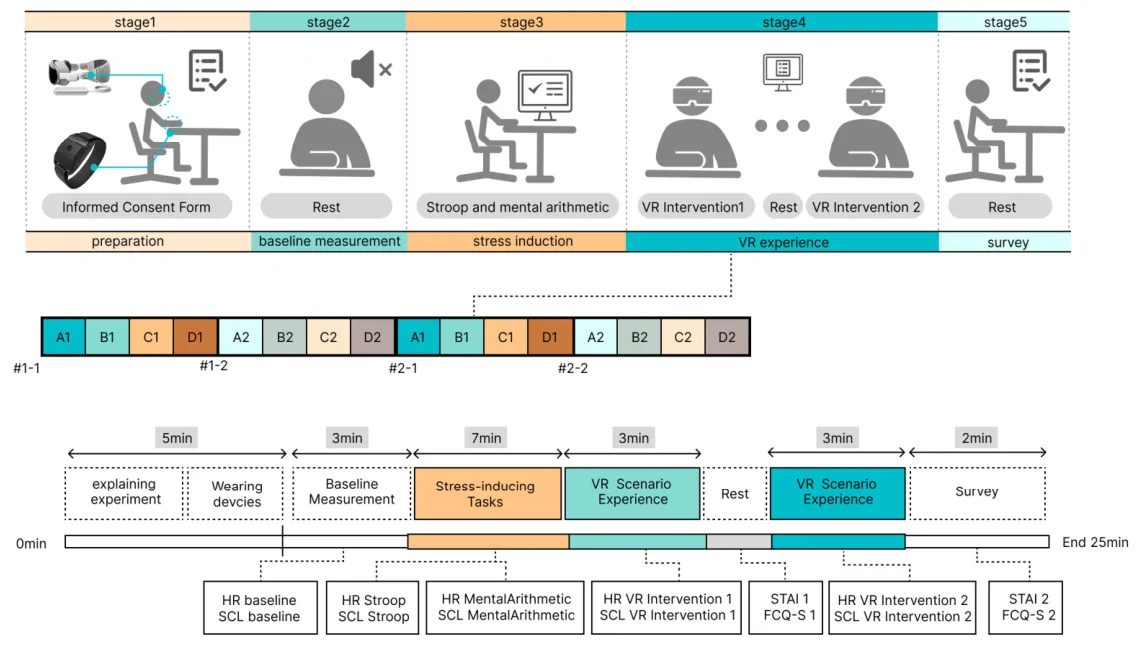
Experimental procedure. Note: HR: Heart Rate, SCL: Skin Conductance Level, STAI: State–Trait Anxiety Inventory, FCQ-S: Food Craving Questionnaire—State.

Each round of the experiment lasted about 27 min and consisted of five stages: (1) preparation; (2) baseline measurement; (3) stress induction using the Stroop task and mental arithmetic; (4) VR experience; (5) survey (Figure 2). Physiological indicators were continuously measured throughout the experiment.

1. Participants were briefed on the experimental procedure, signed an informed consent form, familiarized themselves with the environment, and tested the VR equipment. The day before the experiment, participants completed a food preference questionnaire and a personal information form. If participants experienced any discomfort while wearing the VR equipment, they were asked to report the discomfort and remove the VR equipment.
2. Baseline Measurement: Initially, participants wore the Ergosensing wristband. Before the virtual reality experience, participants underwent a 3-min baseline test to measure their resting and stress levels.
3. Stress-inducing tasks: The participants completed two stress-inducing tasks, the Stroop and mental arithmetic tests, to induce psychological stress responses (Y. Liu et al., 2019; Meng & Zhang, 2025). The stress induction effect was evaluated by comparing physiological data before and after the tasks, offering a basis for subsequent analysis (Kexin et al., 2024).
4. VR scenario experience: Participants first experienced Scene A for 3 min, after which auditory cues were changed while keeping the visual color condition the same, and they then entered Scene B for another 3-min experience. A brief break was given between the two VR experiences to reduce fatigue and minimize sequence effects. The presentation order of the scenes was randomized to ensure balanced control over experimental conditions. All participants experienced both scene orders equally across the sample.
5. Survey: After each scene experience, participants completed an electronic survey, which included the subjective state–trait anxiety inventory (STAI), the short version of the Food Craving Questionnaire—State (FCQ-S), and the Virtual Reality Sickness Questionnaire (VRSQ). These responses were used for further analysis of the regulatory effects of multimodal stimuli on participants’ emotions and food-related responses.

After the experiment, researchers assisted participants in removing the equipment and conducted basic cleaning of the experimental environment.

### 2.5. Outcome Measures

Through the pre-experiment questionnaire, we collected participants’ basic information one day before the experiment, including gender, age, weight, height, and the DEBQ-EES Emotional Eating Questionnaire. The post-experiment questionnaire included the simplified version of the IPQ (Igroup Presence Questionnaire) and the VRSQ (Virtual Reality Sickness Questionnaire) to assess participants’ experiences in the virtual reality environment. Participants who reported dizziness were excluded from the data. During the VR experiment, we collected data on participants’ stress levels and food craving intensity (Table 1).

#### 2.5.1. State–Trait Anxiety Inventory (STAI)

This study used the shortened, validated version of the State–Trait Anxiety Inventory (STAI), a method for measuring stress. It includes 10 questions, with 3 positive and 7 negative items, rated on a 5-point Likert scale (1–5, from “strongly disagree” to “strongly agree”). Positive items are scored reversely, while negative items are scored directly. The average score represents the level of stress. To assess the impact of rest on stress recovery, we administered two STAI tests to calculate the difference in stress levels after two VR scene experiences (X. Gao et al., 2025). 

#### 2.5.2. Skin Conductance Level (SCL) and Heart Rate (HR)

Skin conductance level (SCL) and heart rate (HR) were used as objective measures of stress. These physiological measures reflect sympathetic and parasympathetic nervous system activity, providing a comprehensive assessment of changes in autonomic nervous activity under various audiovisual emotional stimuli (Bian et al., 2022; C. Gao & Zhang, 2020; Lee & Hong, 2025). Among these, SCL is a key indicator of skin conductance activity (GSR). SCL reflects the sustained level of skin conductance over time, usually related to baseline stress or chronic emotional responses. A reduction in SCL indicates increased relaxation, accompanied by decreased stress levels (Gaekwad et al., 2023; X. Zhou et al., 2024). Heart rate (HR) is an important physiological signal for identifying tension, anxiety, or relaxation, with HR increasing under stressful conditions (Abd-Alhamid et al., 2020; Bian et al., 2022). To obtain SCL and HR signals, participants were required to wear customized wristbands (Ergosensing, China) on their non-dominant hand during the experiment (N. Liu et al., 2025; Y. Zhang et al., 2023). 

In the subsequent analysis, data analysis will be performed on the SCL and HR values under different experimental conditions, including comparisons of pre- and post-stimulus differences and interactions between conditions. The goal is to explore the regulatory impact of different types of emotional induction on the autonomic nervous system, thus providing physiological evidence for the design of emotional recognition and intervention strategies. 

#### 2.5.3. Dutch Eating Behavior Questionnaire—Emotional Eating Subscale (DEBQ-EES)

Emotional eating tendencies were assessed using the 13-item emotional eating subscale of the Dutch Eating Behavior Questionnaire (DEBQ-EES) (van Strien et al., 1986). Participants responded based on the frequency of eating in different emotional states before the experiment, using a 5-point scale from “never” (1 point) to “very often” (5 points). Higher scores indicate a stronger tendency toward emotional eating (negative EE). Since emotional eating (EE) follows a continuous distribution among general university students and is relatively common, this study did not pre-screen for EE traits during the recruitment phase. This approach ensures a representative sample and enhances the generalizability of the findings. Following the experiment, participants’ EE tendencies were assessed using the questionnaire, and the sample was categorized into high and low EE tendency groups. This grouping analysis facilitates a more precise understanding of the differences in responses between individuals with varying levels of EE under the intervention of a multimodal sensory environment. Based on previous research, a DEBQ-EES score of 3.25, corresponding to the 80th percentile of Dutch normative samples, was used as the cutoff for identifying individuals with a higher tendency toward emotional eating (Frayn & Knäuper, 2017; Sze et al., 2021). This grouping analysis was exploratory.

#### 2.5.4. Healthy Food Preference Task

**Figure 3:**
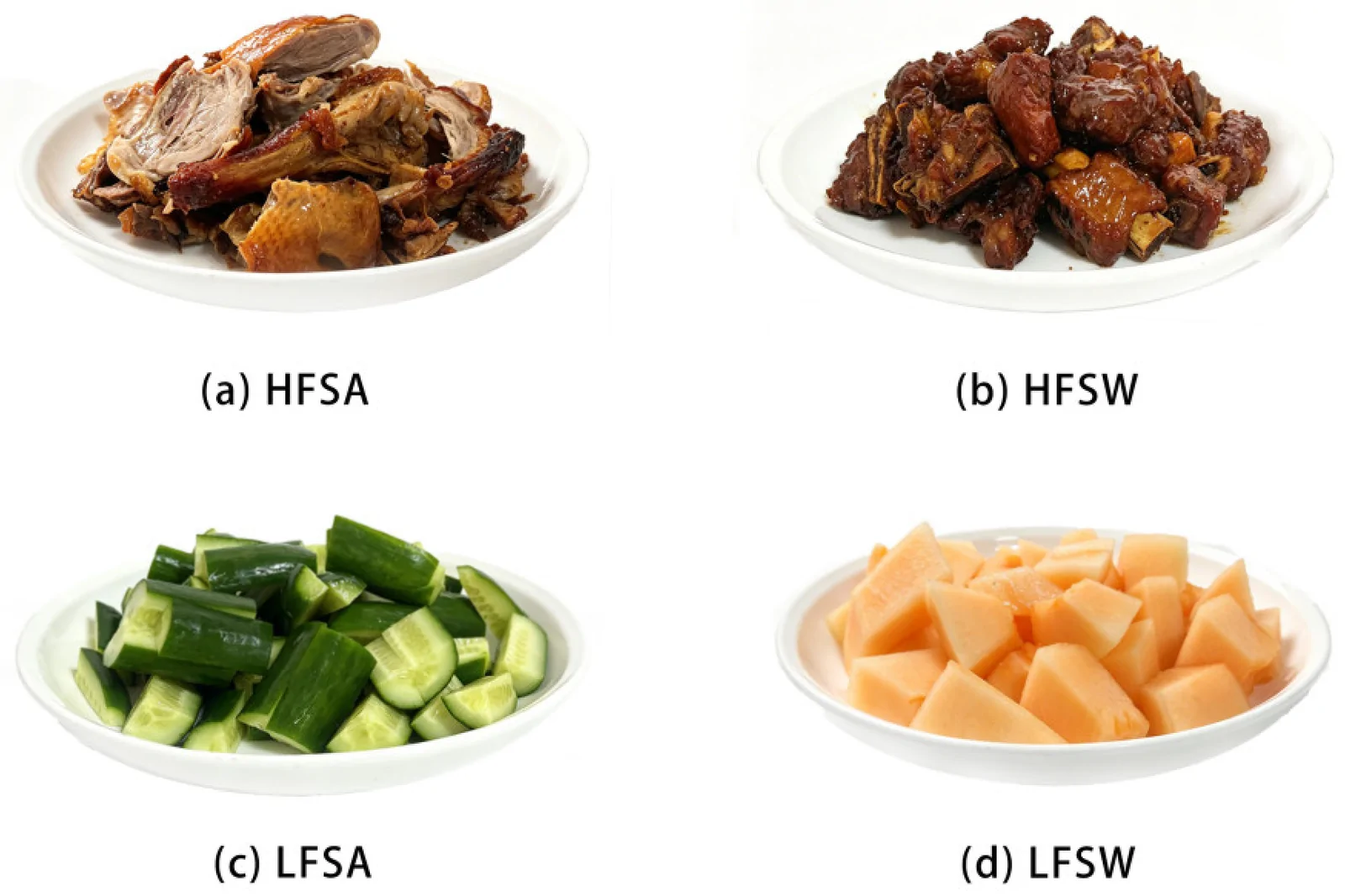
Standard pictures from four categories. (**a**) High-calorie savory (HFSA): roast duck; (**b**) high-calorie sweet (HFSW): sweet and sour pork ribs; (**c**) low-calorie savory (LFSA): cucumber salad; (**d**) low-calorie sweet (LFSW): Hami melon. The images were selected from a previously constructed and validated Chinese food image database (Y. Zhou et al., 2023). Note: HFSA = high-calorie savory; HFSW = high-calorie sweet; LFSA = low-calorie savory; LFSW = low-calorie sweet.

The food image materials used in this study were derived from the previously constructed and verified Chinese food image database (Y. Zhou et al., 2023). Based on prior literature, four representative food categories were selected as experimental stimuli: high-calorie sweet, high-calorie savory, low-calorie sweet, and low-calorie savory foods (Figure 3). Four candidate foods were selected from each category (16 images in total), and a pre-experiment preference rating was conducted. Based on participants’ ratings, one highly preferred food was selected from each category, resulting in four food images for the formal experiment.

The health rating of food was constructed with reference to the methods in existing literature and quantified by subjective evaluation by 7 experts. The nutritional health of each type of food was anonymously and independently rated on a scale of 1–10, with higher scores indicating healthier nutritional structures (see Table S1 of Supplementary Materials) (Talati et al., 2025). Before the experiment, participants were briefly familiarized with the four food categories. During the formal experiment, the four food images were presented simultaneously, and participants selected the food they most wanted to eat. The expert-rated healthiness score of the selected food was used as the healthy food preference score for that trial.

#### 2.5.5. Food Craving Questionnaire—State (FCQ-S)

Food craving intensity was assessed using the “Desire” subscale of the Food Craving Questionnaire—State (FCQ-S) (Shireen et al., 2024). Participants rated these items based on the same four food images presented during the food preference task. In this study, the FCQ-S score ranges from 6 to 15, with higher scores indicating a stronger desire to eat. This study used three core items, including “How much do you crave one or more foods?” “How intense is this craving?” and “How strong is your urge to eat this food?”. The rating scale ranged from 1–5, with higher scores indicating stronger cravings (see Supplementary Table S2).

### 2.6. Data Statistical Analysis

The statistical analyses consisted of two parts: primary experimental effect analyses and exploratory individual-difference analyses. For the primary analyses, a mixed-design repeated-measures ANOVA was employed, with visual color (between-subjects factor, four levels) and ambient sound (within-subjects factor, two levels) as independent variables, to examine their main effects and interaction on the psychological and physiological outcome measures. Before the primary analyses, the assumptions of normality, homogeneity of variance, and the presence of outliers were assessed. Normality was evaluated using the Shapiro–Wilk test and Q–Q plots. Although some deviations from normality were observed for the healthy food choice scores, parametric analyses were retained given the robustness of ANOVA to moderate deviations from normality and the relatively similar group sizes. Levene’s test indicated homogeneity of variance for all dependent variables (*p* > 0.05). Potential outliers were screened using boxplots (1.5 × IQR), and no extreme outliers requiring exclusion were identified. Because the within-subjects factor had only two levels, the sphericity assumption was automatically satisfied, and no correction was required.

For significant main effects, pairwise comparisons were conducted with Bonferroni correction. For significant interactions, simple-effects analyses were further performed to examine the effect of one factor at each level of the other factor, with Bonferroni correction applied to control the family-wise error rate (FWER) within each family of comparisons. 

Exploratory individual-difference analyses included independent-samples *t*-tests and Pearson correlation analyses. Independent-samples *t*-tests were used to examine group differences in psychological responses and food craving according to gender and emotional eating tendency, with emotional eating categorized using the DEBQ-EES cutoff of 3.25. To assess the robustness of the findings to this categorization and minimize information loss associated with dichotomization, DEBQ-EES was additionally treated as a continuous variable using the original mean score, and Pearson correlations were computed with the outcome measures (STAI, FCQ-S).

#### 2.6.1. Data Preprocessing

To assess the regulatory effects of the multisensory virtual reality environment on participants’ stress and autonomic nervous activity, this study collected and processed two types of physiological signals: Galvanic Skin Response (GSR) and Photoplethysmography (PPG). GSR signals were sampled at 4 Hz, and PPG signals at 100 Hz. All signal collection was performed in real time throughout the experiment. Each participant’s data file contained phase markers, which divided the data into five stages. The phase boundaries were determined by the first and second occurrence of the markers, and if the marker information was missing or incomplete, the data from that phase was automatically excluded. 

The GSR signals were scaled to μS (1/10,000 of the original units) and filtered with a 0.8 Hz low-pass Butterworth filter to remove high-frequency noise while preserving valid fluctuations. Subsequently, SCL-related indicators were extracted from the signals of each phase to reflect the level of autonomic nervous activation during each phase. The processing of the PPG signals involved applying a third-order band-pass Butterworth filter with a range of 0.6–5 Hz to suppress motion artifacts and respiratory interference effectively, and Mean HR was subsequently calculated for statistical analyses (Y. Zhang et al., 2023). During physiological data preprocessing, anonymized group codes were used, and investigators were blinded to the specific visual and auditory conditions. Experimental-condition information was revealed only during the subsequent statistical analyses.

#### 2.6.2. Statistical Analysis of Physiological Indicators

To assess whether the VR intervention effectively triggered autonomic nervous system responses, a paired-sample *t*-test was used to compare the differences in key physiological indicators, including mean skin conductance (M SCL) and mean heart rate (M HR), before and after stress induction. The significance level was set at *α* = 0.05 for statistical analysis. This analysis aimed to assess whether the VR intervention significantly altered physiological states, thus validating its effectiveness in inducing stress responses. 

To explore the interaction between visual (color) and auditory (sound) factors, mixed-model ANOVAs were used with color and sound type as fixed factors, analyzing their main and interaction effects on multiple recovery-related physiological indicators (e.g., HR, SCL recovery rate). For variables with significant interaction effects, further simple effect analyses were performed to compare recovery differences under various sound conditions for the same color, with Cohen’s *d* calculated to measure effect size.

#### 2.6.3. Statistical Analysis of Psychological Indicators

This study employed IBM SPSS Statistics 29.0.1.0 to conduct statistical analysis on the experimental data, including variables such as psychological indicators, emotional eating tendency, stress states, food preference, and food craving intensity. First, reliability testing was conducted on the self-report scales used. The results indicated that the DEBQ-EES (Cronbach’s *α* = 0.899), STAI1 (Cronbach’s α = 0.894), STAI2 (Cronbach’s *α* = 0.883), FCQ-S1 (Cronbach’s *α* = 0.855), and FCQ-S2 (Cronbach’s *α* = 0.858) all exhibited high internal consistency (Cronbach’s *α* values greater than 0.7), demonstrating that the scales were reliable in this study. Psychological outcomes were analyzed using the mixed-design ANOVA framework described in Section 2.6. Statistical significance was set at *α* = 0.05.

## 3. Results

The demographic characteristics of the final sample (*n* = 49) are described in detail in Section 2 (Section 2.3). Briefly, the sample consisted of 26 males and 23 females, with an age range of 22–28 years (*M* = 22.43, *SD* = 3.19). Most participants were undergraduate or graduate students.

### 3.1. Effectiveness of Stress Induction

Mean heart rate can serve as a reliable physiological indicator for assessing stress responses (Cook et al., 2025). The stress induction procedure significantly increased HR. Specifically, the HR of participants significantly increased following the stress induction tasks compared with the baseline condition (*t* = 2.515, *p* = 0.0179, Cohen’s *d* = 0.40), indicating that the stress induction tasks effectively elicited a physiological stress response (Table 2).

### 3.2. Effect of Environmental Characteristics on the Recovery of Physiological Indicators

The multisensory VR environment significantly reduced physiological stress indicators (Lattimore, 2020). Specifically, HR significantly decreased during the VR1 phase compared with the stress phase, with a mean reduction of 4.44 bpm (95% CI [−7.52, −1.36], *t* = −2.98, *p* = 0.0079, Cohen’s *d* = −0.67). Heart rate also significantly decreased during the VR2 phase compared with the stress phase, with a mean reduction of 5.54 bpm (95% CI [−8.66, −2.42], *t* = −3.69, *p* = 0.0015, Cohen’s *d* = −0.83). These findings suggest that the multisensory VR environment helped reduce cardiovascular arousal following stress induction. To further examine the effects of different sensory cues on physiological recovery, we calculated a Recovery Index (RI), defined as:

$$Recovery\;Index={Mean}_{stress}-{Mean}_{recovery}$$

The stress value was calculated from the last 20 s of the stress induction task, and the recovery value was calculated from the last 20 s of each VR intervention phase. A higher RI indicates a greater decrease in the physiological measure from the stress level to the recovery phase and therefore represents a greater degree of physiological recovery. A higher RI indicates a greater decrease from the stress level and therefore a greater degree of physiological recovery. We further employed a mixed-design repeated-measures ANOVA to investigate the effects of visual color and auditory condition (spring water sound and indoor environmental background sounds) factors on physiological recovery indicators. For the SCL recovery index, the main effect of vision was significant (*F* = 4.52, *p* = 0.015). The main effect of auditory was not significant (*F* = 0.104, *p* = 0.749). However, a significant interaction between vision and auditory (*F* = 3.92, *p* = 0.029), indicating that the effect of sound on SCL recovery is modulated by visual color. In the Mean HR recovery index, the main effect of vision was also significant (*F* = 2.804, *p* = 0.0423). Although the main effect of auditory was not significant (*F* = 1.917, *p* = 0.1740), the interaction between vision and auditory was significant (*F* = 6.989, *p* = 0.001), further indicating that the effect of sound on HR recovery depends on the visual background. 

Simple effects analysis further clarified the visual–auditory interactions. Under the red background, indoor background sounds yielded higher recovery indices than spring water sounds for both SCL (*M* = 0.257 vs. 0.012, *p* = 0.032) and HR (*M* = 0.058 vs. −0.046, *p* < 0.001). Under the green background, the pattern reversed: spring water sounds yielded higher recovery indices for both SCL (*M* = 0.452 vs. 0.282, *p* = 0.046) and HR (*M* = 0.121 vs. 0.054, *p* < 0.001). Under the white background, spring water sounds yielded a higher HR recovery index than indoor background sounds (*M* = −0.012 vs. −0.044, *p* = 0.014), whereas no significant auditory differences were observed under the blue background for either measure (*p* > 0.05) (Table 3).

### 3.3. The Influence of Environmental Characteristics on Food Preference

Participants’ preference for healthier food options for healthier options was assessed using an image-selection task. The results of the mixed-design ANOVA showed that sound type (*F* = 36.023, *p* < 0.001, *η*^2^ = 0.445) and color (*F* = 6.130, *p* = 0.001, *η*^2^ = 0.290) had significant main effects on healthy food preference scores, and there was a significant interaction between the two (*F* = 3.584, *p* = 0.021, *η*^2^ = 0.193). Table 4 presents the healthy food preference scores under the different environmental conditions. Simple-effects analyses showed that spring water sounds significantly increased healthy food preference scores compared with indoor background sounds under the red (*M* = 7.782 vs. 3.573, *p* < 0.001, Cohen’s *d* = 1.25), blue (*M* = 9.206 vs. 7.604, *p* = 0.021, Cohen’s *d* = 0.58), and green (*M* = 9.273 vs. 7.344, *p* = 0.017, Cohen’s *d* = 0.65) backgrounds, with the largest mean difference observed under the red background. Under the white background, healthy food preference scores were also higher under spring water sounds, but the difference was not statistically significant (*p* = 0.122). 

### 3.4. The Influence of Environmental Characteristics on Psychological Indicators

A mixed-design ANOVA revealed a significant main effect of color (*F* = 6.149, *p* = 0.001, *η*^2^ = 0.291), a significant main effect of sound (*F* = 49.722, *p* < 0.001, *η*^2^ = 0.525), and a significant interaction between color and sound (*F* = 5.087, *p* = 0.004, *η*^2^ = 0.253). Simple-effects analyses showed that spring water sounds were associated with lower STAI scores than indoor background sounds under the blue (*M* = 18.857 vs. 25.857, *p* < 0.001), green (*M* = 15.300 vs. 26.200, *p* < 0.001), and white (*M* = 27.167 vs. 31.167, *p* = 0.022) backgrounds, whereas no significant difference was observed under the red background (*M* = 27.385 vs. 29.231, *p* = 0.261) (Table 4).

### 3.5. The Influence of Environmental Characteristics on Food Craving

The Food Craving Questionnaire—State (FCQ-S) measures subjective motivation to eat, serving as a proximal indicator of EE propensity activation. The mixed-design ANOVA results indicated that the color environment significantly influenced the FCQ-S score (*F* = 3.975, *p* = 0.014, *η*^2^ = 0.209); the sound background also had a significant impact (*F* = 11.795, *p* < 0.001, *η*^2^ = 0.208). However, the interaction between color and sound approached but did not reach significance (*F* = 2.432, *p* = 0.077, *η*^2^ = 0.140). Further descriptive analysis showed that blue and green environments were associated with lower FCQ-S scores, suggesting a potential appetite-suppressing effect. This trend was consistent across both sound conditions. Moreover, spring water sounds were associated with lower FCQ-S scores than indoor background sounds under the red (*M* = 10.000 vs. 11.538, *p* < 0.001) and blue (*M* = 9.429 vs. 10.643, *p* = 0.006) backgrounds, whereas no significant differences were observed under the green or white backgrounds (Table 4).

### 3.6. Gender and DEBQ-EES Differences in Outcome Indicators

This study further examined differences in outcome indicators according to gender and emotional eating tendency. Significant between-group differences were observed for several psychological and food-related outcomes.

Significant between-group differences were observed according to gender for emotional responses (STAI1, *p* = 0.003; STAI2, *p* = 0.006) and food craving (FCQ-S1, *p* < 0.001; FCQ-S2, *p* < 0.001). Additionally, female participants showed higher mean scores in both STAI (STAI1: females, *M* = 31.30; males, *M* = 25.31; STAI2: females, *M* = 25.52; males, *M* = 19.69) and FCQ-S (FCQ-S1: females, *M* = 12.87; males, *M* = 9.58; FCQ-S2: females, *M* = 12.04; males, *M* = 8.73) compared to male participants (Table 5).

The between-group differences in emotional responses varied across the two STAI assessments. No significant difference was observed between the high- and low-EE groups for STAI1 (*p* = 0.195), whereas a significant difference was observed for STAI2 (*p* = 0.022). Similarly, significant between-group differences were observed for food craving. The high-EE group reported higher scores than the low-EE group on both FCQ-S1 (high EE: *M* = 12.75; low EE: *M* = 10.59, *p* = 0.008) and FCQ-S2 (high EE: *M* = 12.17; low EE: *M* = 9.68, *p* = 0.003) (Table 5). In the exploratory analyses, DEBQ-EES scores were positively correlated with FCQ-S2 (*r* = 0.348, *p* = 0.014), whereas the correlations with FCQ-S1 (*r* = 0.266, *p* = 0.064), STAI1 (*r* = 0.205, *p* = 0.158), and STAI2 (*r* = 0.151, *p* = 0.299) were not statistically significant.

## 4. Discussion

This study used VR technology to examine how combinations of visual color and environmental sound may influence physiological recovery, psychological states, food preferences, and subjective food craving. The findings provide preliminary evidence for understanding the potential role of multisensory environmental design in stress recovery and food-related responses.

### 4.1. Associations of Visual Environment with Physiological and Psychological Recovery, Food Craving, and Healthy Food Preference

The observed effects of wall color are broadly consistent with previous research linking cool-colored environments with lower physiological arousal and greater relaxation (Bower et al., 2022; Weijs et al., 2023). Blue and green environments may promote a calmer physiological state, whereas red is more commonly associated with heightened physiological activation. 

The stronger emotional recovery observed under blue and green backgrounds may be related to their common associations with safety and calmness. The relatively large effect of color on STAI scores further suggests that visual color may have a meaningful contribution to subjective emotional recovery. In contrast, red is often linked to danger and warnings, which may trigger psychological stress. This finding aligns with existing research on color psychology and emotion regulation.

The association between visual color and subjective food craving is consistent with previous findings that color can influence food-related responses (Singh, 2006; Suk et al., 2012; Szocs et al., 2024). In the present study, cool-toned environments were associated with lower subjective food craving. Notably, the red background also showed lower food craving, which may be related to psychological responses to color, as red can evoke a sense of caution or alertness (Genschow et al., 2012).

The association between visual color and healthy food preference is broadly consistent with previous research (Songur Bozdag & Akkurt, 2025). In the present study, blue and green backgrounds were associated with higher healthy food preference scores, whereas red backgrounds were associated with lower scores. These findings suggest that cool-colored environments may be related to differences in cognitive food preference, although the underlying mechanisms require further investigation.

### 4.2. Associations of Auditory Environment with Physiological and Psychological Recovery, Food Craving, and Healthy Food Preference

Previous research has suggested that natural sounds, including spring water sounds, may reduce subjective stress and support psychological well-being (C. Liu et al., 2024; Peng-Li et al., 2022; N. Zhang et al., 2025). However, in the present study, the main effects of sound on SCL and HR were not statistically significant. This suggests that the physiological responses to auditory conditions may depend on the visual context in this experimental setting.

The stronger physiological recovery associated with spring water sounds in some visual conditions is broadly consistent with previous research. The relatively large effect of sound on STAI scores indicates that auditory conditions may have a substantial association with subjective emotional responses in this experimental setting. However, this pattern was reversed under the red background, where indoor background sounds were associated with a higher HR recovery index than spring water sounds. This contrast highlights the context-dependent nature of auditory effects on physiological recovery.

Spring water sounds were associated with lower STAI scores than indoor background sounds, consistent with previous research suggesting that natural sounds may support psychological well-being. However, this association was not significant under the red background, suggesting that the psychological effects of auditory conditions may depend on the visual context.

The lower FCQ-S scores observed with spring water sounds are consistent with previous findings, suggesting that natural sounds may be associated with lower subjective food craving (Phillips & Rech, 2026). This association was most pronounced under the red background. Similarly, spring water sounds were associated with higher healthy food preference scores, consistent with previous research suggesting that natural sounds may influence food-related preferences.

Overall, these findings suggest that natural sounds may contribute to psychological and food-related responses, while their effects on physiological recovery may depend on the accompanying visual environment. Such context-dependent responses may be related to differences in the integration of visual and auditory cues (R. Liu et al., 2019). 

### 4.3. Visual and Auditory Environment Interaction Effects on Physiological and Psychological Recovery, Food Craving, and Healthy Food Preference

The significant interactions between visual and auditory conditions across physiological and psychological outcomes indicate that the effects of auditory stimulation depended on the visual context. The direction and magnitude of auditory effects varied across color conditions, and this pattern was particularly evident under the red and green backgrounds. Such context-dependent responses may be related to differences in the integration of visual and auditory cues (Gie Liem et al., 2023; Kong & Han, 2024). 

Psychologically, colors and sounds also exhibit significant interactions. The combination of green and blue visual environments with spring water sound was more effective in alleviating negative emotions than the white visual environment, suggesting that cool tones and natural sounds may be beneficial for emotional recovery. Overall, the joint stimulation of vision and hearing shows conditional dependence, supporting the potential role of multisensory integration in emotional and physiological recovery.

For food craving, however, the color × sound interaction did not reach statistical significance, despite significant main effects of both color and sound. This suggests that the effects of color and sound on subjective food craving were not clearly dependent on their combination. In contrast, a significant color × sound interaction was observed for healthy food preference, indicating that the effect of sound on food preference varied across color conditions. These findings further suggest that visual and auditory environmental cues may influence different food-related responses in distinct ways.

### 4.4. Individual Differences in Emotional and Food-Craving Responses

Gender differences were observed in emotional and food-craving responses, with female participants showing higher STAI and FCQ-S scores than male participants. This finding is broadly consistent with previous research suggesting greater susceptibility to stress-related food responses among women (Houminer Klepar et al., 2024; Marchena-Giraldez et al., 2024). However, the evidence on gender differences is not entirely consistent, suggesting that such differences may vary across populations and contexts (Margherita, 2015). 

Between-group differences according to emotional eating tendency were observed in several outcome measures, with participants in the high emotional-eating tendency group showing higher anxiety and food-craving scores following stress than those in the low emotional-eating tendency group. This pattern is consistent with previous research suggesting that individuals with greater emotional eating tendencies may be more responsive to environmental and emotional cues (Chawner & Filippetti, 2024). The exploratory continuous analysis provided additional evidence of a positive association between emotional eating tendency and food craving, as DEBQ-EES scores were positively correlated with FCQ-S2 scores.

### 4.5. Strengths and Limitations

This study expands the application of the EOH model in VR environments, highlighting the potential role of environmental sensory elements in stress recovery and food-related responses. The findings provide empirical evidence for understanding how multisensory environmental cues may influence physiological, psychological, and food-related responses. The combination of cool tones and natural sounds may represent a non-invasive approach worthy of further investigation. Additionally, the use of VR provides a controlled experimental environment for examining multisensory environmental effects and may offer methodological insights for future digital health research.

Although this study demonstrates theoretical and methodological contributions within its current scope, it also has certain limitations. First, the sample size was limited (*n* = 49), and participants were recruited from a single Chinese university and were predominantly graduate students. This may limit the generalizability of the findings to populations with different cultural and dietary backgrounds and reduce statistical power for detecting small effects, higher-order interactions, and subgroup differences. 

Second, the controlled experimental design may limit ecological validity (Rosa et al., 2026; van den Bogerd et al., 2020). Participants remained seated and were exposed to each virtual environment for only three minutes, which differs from typical real-world eating situations. The two-hour fasting requirement may also have resulted in differences in hunger levels. In addition, only two auditory stimuli were examined, and olfactory and tactile stimuli were not included. Individual differences and environmental factors such as temperature were not fully controlled. Although physiological responses were used to validate the stress induction procedure, comprehensive pre- and post-stress psychological measures were not included.

Third, actual food intake was not directly measured. Instead, food-related responses were assessed using image-based food preferences and subjective food craving measures. Thus, the findings reflect changes in food-related responses rather than actual eating behavior. Future research should incorporate objective behavioral measures, such as laboratory-based dietary tasks and quantitative assessment of food intake.

Finally, multiple statistical comparisons across physiological, psychological, and food-related outcomes may have increased the risk of Type I error. The emotional eating tendency score was also dichotomized based on a predefined cutoff, which may have reduced statistical sensitivity. An exploratory continuous analysis was additionally conducted to examine associations between DEBQ-EES scores and the outcome measures. In addition, mediation effects among environmental exposure, stress recovery, and food-related responses were not tested. Future studies should retain emotional eating tendency as a continuous variable in confirmatory analyses and use mediation or structural modeling approaches to further examine potential mechanisms.

## 5. Conclusions

This study provides preliminary evidence that multisensory environmental cues may be associated with stress recovery and food-related responses among college students. Within the virtual reality environment, cool-toned visual environments and natural sounds were associated with greater physiological recovery and improved emotional states, as well as higher image-based preferences for healthier foods and lower subjective food craving. These findings highlight the potential relevance of multisensory environmental cues to stress recovery and food-related responses, while the conditions under which these associations occur and their underlying mechanisms require further investigation.

Future research should use larger and more diverse samples and longer-term designs to examine the generalizability and persistence of these findings. Studies could also incorporate additional sensory modalities, objective measures of food intake, and individual psychological traits to further examine multisensory environmental effects and individual differences in stress recovery and food-related responses. The feasibility of translating these findings into real-world or digital health applications also requires further empirical validation.

## Figures and Tables

**Table 1:** Summary of outcome measures.

EOH	Category	Measure
Environment	sensory environmental cues	Visual environmental cues (Color)
		Auditory environmental cues (Sound)
Occupant	subjective pressure	State–Trait Anxiety Inventory (STAI) Stress
	objective pressure	Skin Conductance Level (SCL)
		Heart Rate (HR)
Health/Behavior	healthier food preferences	Healthy Food Preference Score
	food craving	Food Craving Questionnaire—State (FCQ-S)

**Table 2:** Physiological responses to stress induction.

Stages	Mean HR	Mean Difference (Stress − Baseline)	95% CI	*t*	*p*-Value	Cohen’s *d*
baseline	73.13 ± 9.02	2.15	[0.61, 3.69]	2.515	0.0179	0.40
stress induction	75.28 ± 8.70					

Note: Mean HR = mean heart rate; CI = confidence interval; Cohen’s *d* = effect size. Mean difference = Stress − Baseline, with positive values indicating an increase and negative values indicating a decrease.

**Table 3:** Simple effects analysis of noise and spring water on SCL and HR recovery index under different color conditions in vision × sound interaction.

Measure	Condition	Indoor Background Sounds (*M*)	Spring Water (*M*)	*p*-Value	Cohen’s *d*
SCL	Red	0.257	0.012	0.032 *	0.944
	Blue	0.431	0.425	0.954	0.021
	Green	0.282	0.452	0.046 *	−0.857
	White	0.522	0.547	0.749	0.117
HR	Red	0.058	−0.046	<0.001 ***	1.787
	Blue	−0.018	0.068	0.069	−0.555
	Green	0.054	0.121	<0.001 ***	−1.875
	White	−0.044	−0.012	*p* = 0.014 *	0.966

Note: *M* = Mean; SCL = Skin Conductance Level; HR = Heart Rate. * *p* < 0.05; *** *p* < 0.001 based on unadjusted *p*-values. Bonferroni correction was applied for multiple comparisons; After correction, the sound effects on HR remained significant under the red and green background conditions.

**Table 4:** Effects of color and sound conditions on healthy food choice, STAI, and FCQ-S scores.

Measure	Condition	Indoor Background Sounds (I)		Spring Water (J)		Mean Change (I-J)	*SE*	*p*-Value
		Mean	95% CI [LL, UL]	Mean	95% CI [LL, UL]			
Healthy food preference	Red	3.573	[1.996, 5.150]	7.782	[6.997, 8.568]	−4.209	0.714	<0.001 ***
	Blue	7.604	[6.084, 9.123]	9.206	[8.449, 9.963]	−1.602	0.688	0.021 *
	Green	7.344	[5.546, 9.142]	9.273	[8.377, 10.169]	−1.929	0.814	0.017 *
	White	6.752	[5.111, 8.393]	7.907	[7.089, 8.724]	−1.155	0.743	0.122
STAI	Red	29.231	[25.243, 33.219]	27.385	[24.131, 30.638]	1.846	1.622	0.261
	Blue	25.857	[22.014, 29.700]	18.857	[15.722, 21.992]	7.000	1.563	<0.001 ***
	Green	26.200	[21.653, 30.747]	15.300	[11.590, 19.010]	10.900	1.849	<0.001 ***
	White	31.167	[27.016, 35.317]	27.167	[23.780, 30.553]	4.000	1.688	0.022 *
FCQ-S	Red	11.538	[10.243, 12.834]	10.000	[8.661, 11.339]	1.538	0.435	<0.001 ***
	Blue	10.643	[9.395, 11.891]	9.429	[8.138, 10.719]	1.214	0.419	0.006 **
	Green	9.500	[8.023, 10.977]	9.400	[7.873, 10.927]	0.100	0.496	0.841
	White	12.583	[11.235, 13.931]	12.333	[10.940, 13.727]	0.250	0.453	0.584

Note: STAI = State–Trait Anxiety Inventory; FCQ-S = Food Craving Questionnaire—State; *SE* = Standard Error; CI = Confidence Interval; UL = Upper Limit; LL = Lower Limit. * = *p* < 0.05; ** = *p* < 0.01; *** = *p* < 0.001 based on unadjusted *p*-values. Bonferroni correction was applied to account for multiple comparisons, and Bonferroni-adjusted significance was considered when interpreting the results.

**Table 5:** Independent-samples *t*-test results for gender and DEBQ-EES-related group differences.

Grouping Variable	Measure	Group 1 (*M*)	Group 2 (*M*)	Cohen’s *d*	*p*-Value
Gender		Male	Female		
	STAI1	25.31	31.30	−0.899	0.003 **
	STAI2	19.69	25.52	−0.818	0.006 **
	FCQ-S1	9.58	12.87	−1.747	<0.001 ***
	FCQ-S2	8.73	12.04	−1.630	<0.001 ***
DEBQ-EES		Low DEBQ-EES (<3.25)	High DEBQ-EES (≥3.25)		
	STAI1	27.35	30.50	−0.437	0.195
	STAI2	21.03	26.75	−0.784	0.022 *
	FCQ1	10.59	12.75	−0.921	0.008 **
	FCQ2	9.68	12.17	−1.036	0.003 **

Note: *M* = Mean; STAI1 = State–Trait Anxiety Inventory after VR Intervention 1; STAI2 = State–Trait Anxiety Inventory after VR Intervention 2; FCQ-S1 = Food Craving Questionnaire—State after VR Intervention 1; FCQ-S2 = Food Craving Questionnaire—State after VR Intervention 2; DEBQ-EES = Dutch Eating Behavior Questionnaire—Emotional Eating Subscale; Cohen’s *d* = effect size. * = *p* < 0.05; ** = *p* < 0.01; *** = *p* < 0.001, based on unadjusted *p*-values.

## Data Availability

The original contributions presented in the study are included in the article; further inquiries can be directed to the corresponding author.

## Supplementary Materials

The following supporting information can be downloaded at: https://www.mdpi.com/article/10.3390/bs16091471/s1, Table S1: Beverage Healthiness Rankings; Table S2. Food Craving Questionnaire—State (FCQ-S) Items and Subscales; Figure S1: Mean and Standard Deviation of Heart Rate (HR) Across Stages.

## Abbreviations

EE	Emotional Eating
EOH	Environment–Organism–Health
STAI	State–Trait Anxiety Inventory
SCL	Skin Conductance Level
HR	Heart Rate
FCQ-S	Food Craving Questionnaire—State
SPSS	Statistical Package for the Social Sciences
DEBQ-EES	Dutch Eating Behavior Questionnaire—Emotional Eating Subscale
