# Supplementary material for: Effects of Multisensory Environmental Cues on Food Craving, Healthy Food Preference, and Stress-Related Recovery
Source: Behav Sci (Basel). 2026 Aug 24;16(9):1471. doi: 10.3390/bs16091471 (PMC13603129; doi:10.3390/bs16091471)

Supplementary Materials

Table S1. Beverage Healthiness Rankings

Category	Final Ranking	Rating Scores		
		Range	Mean	Median
Roast duck	1	1–3	2.14	2
Sweet and Sour Ribs	2	1–2	1.29	1
Cucumber in sauce	3	7–9	7.86	8
Hami melon	4	9–10	9.43	9

Table S2. Food Craving Questionnaire-State (FCQ-S) Items and Subscales

Subscale	Item	Item Content
An intense desire to eat	1	I have an intense desire to eat [target Foods].
	2	I have a craving for [target Foods].
	3	I have an urge to eat [target Foods].
Anticipation of positive reinforcement	4	Eating [targetFoods] would make things seem just perfect.
	5	I would be very happy if I could eat [target Foods] right now.
	6	I would feel more alert if I could eat [target Foods] right now.
Anticipation of relief from negative states	7	If I ate something, I wouldn't feel so sluggish and lethargic.
	8	I would feel more energetic if I could eat [target Foods] right now.
	9	If I could eat [target Foods] right now, I would feel less tense.
Lack of control over eating	10	My desire to eat [target Foods] seems overpowering.
	11	I know I'm going to keep on thinking about [target Foods] until I actually have it.
	12	If I had [targetFoods] right now, I would not be able to stop eating it.
Craving as a physiological state	13	I am hungry.
	14	My stomach feels empty.
	15	I feel weak because I haven't eaten.

Note: [targetFoods] refers to the specific food(s) the participant is craving at the time of assessment. Responses are scored on a 5-point Likert scale ranging from 1 (strongly disagree) to 5 (strongly agree). Subscale scores range from 3 to 15; total scores range from 15 to 75, with higher scores indicating more intense state food craving [Cepeda-Benito et al., 2000].

Figure s1. Mean and Standard Deviation of Heart Rate (HR) Across Stages

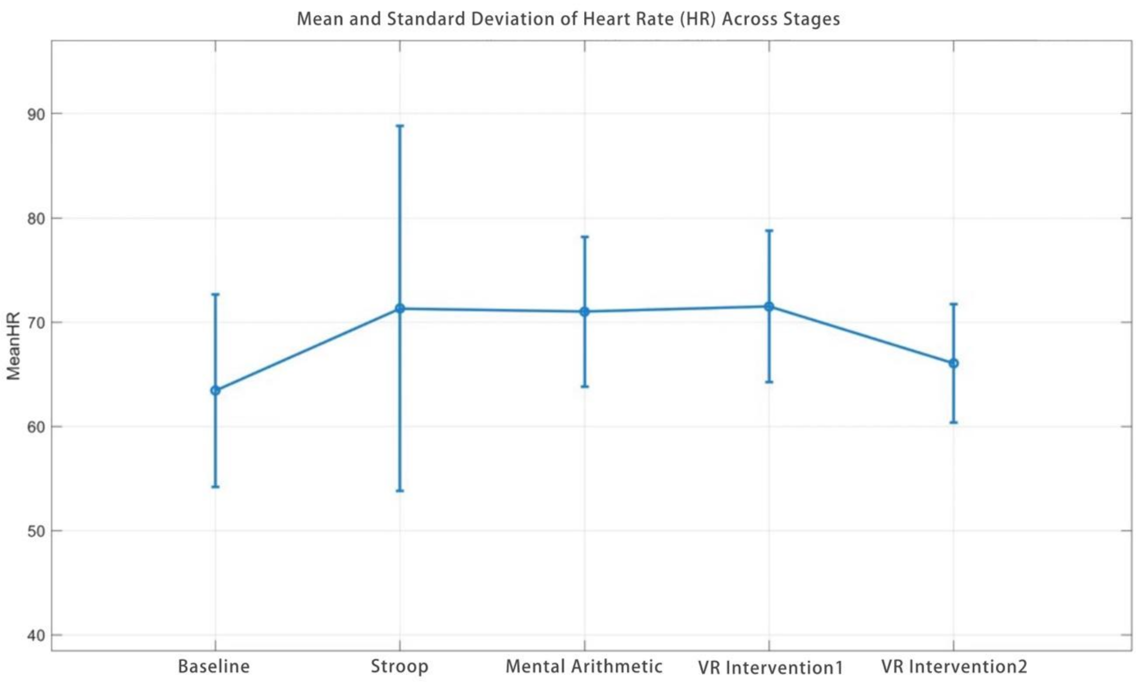

Supplement: Supplementary file 1 [file behavsci-16-01471-s001.zip › behavsci-4429189-supplementary.pdf]
